# On the Treatment of Empyæmia

**Published:** 1893-11

**Authors:** 


ON THE TREATMENT OF EMPYEMA.—In the *British Medical Journal* of July 22, 1893, Chapman gives his views on the treatment of empyema. He has operated with success in seventeen case. The spot for incision should be determined by the peculiarities of the case. As there is always a preliminary puncture over the maximum seat of dullness, the incision should also be over this region, to include the puncture. Both should be made as far back as possible. In practice he finds the spot situated, most often, at about the upper edge of the ninth and tenth ribs, in a line with the angle of the scapula. By two strokes of the knife the chest is opened, and two fingers inserted as far as possible. He advises the insertion of a finger once a week during the subsequent dressings, even if all appears to be going on well. Counter openings have never been found necessary. Resection of a rib he believes unnecessary, but in an extreme case a simple division of one is performed. The only satisfactory drainage is a simpe one, three or four inches long with thick walls, it should have attached to it a shield, two and a half inches square, and should be held in position by four tapes, two around the body and two over the opposite shoulder. The discharge is received in salembroth wool.—*Ex.*

